# New Coleoptera records from New Brunswick, Canada: Eucinetidae and Scirtidae

**DOI:** 10.3897/zookeys.179.2580

**Published:** 2012-04-04

**Authors:** Reginald P. Webster, Jon D. Sweeney, Ian DeMerchant

**Affiliations:** 1 Natural Resources Canada, Canadian Forest Service - Atlantic Forestry Centre, 1350 Regent St., P.O. Box 4000, Fredericton, NB, Canada E3B 5P7

**Keywords:** Eucinetidae, Scirtidae, Scirtoidea, Canada, New Brunswick, new records

## Abstract

We report two species of Eucinetidae, *Nycteus oviformis* (LeConte) and *Nycteus punctulatus* (LeConte), new to New Brunswick, Canada and confirm the presence of *Nycteus testaceus* (LeConte). *Nycteus oviformis* is newly recorded from the Maritime provinces. Additional locality data are provided for *Eucinetus haemorrhoidalis* (Germar) and *Eucinetus morio* LeConte. Five species of Scirtidae, *Cyphon ruficollis* (Say),*Prionocyphon discoideus* (Say), *Sacodes pulchella* (Guérin-Méneville), *Elodes maculicollis* Horn, and *Sarabandus robustus* (LeConte) are added to the New Brunswick faunal list. *Sarabandus robustus* is newly recorded from Canada; *Cyphon ruficollis*, *Prionocyphon discoideus* and *Sacodes pulchella* are new for the Maritime provinces. Collection and habitat data, and distribution maps are presented for these species.

## Introduction

This paper treats new records from New Brunswick of two related families of beetles, the Eucinetidae and Scirtidae. The Eucinetidae of the Maritime provinces (New Brunswick, Nova Scotia, Prince Edward Island) of Canada was recently treated by Majka (2010), who reported two species from New Brunswick. Campbell (1991a) reported seven species of Scirtidae from New Brunswick. However, there have been no recent treatments of this family from the region. Intensive sampling in New Brunswick by the first author since 2003 and records obtained from by-catch samples from Lindgren funnel traps in various New Brunswick forest habitats from 2008–2011 have yielded additional new provincial records in the above families. The purpose of this paper is to report on these new records. A brief synopsis of each family is included in the results below.

## Methods and conventions

The following records are based on specimens collected during a general survey by the first author to document the Coleoptera fauna of New Brunswick and from by-catch samples obtained in trapping experiments testing attractants for surveying Cerambycidae. Additional provincial records were obtained from specimens contained in the collection belonging to Natural Resources Canada, Canadian Forest Service - Atlantic Forestry Centre, Fredericton, New Brunswick.

### Collection methods

Various methods were employed to collect the species reported in this study. Details are outlined in Webster et al. (2009, Appendix). Many specimens were also collected from Lindgren 12-unit funnel trap samples. These traps mimic tree trunks and are often effective for sampling species of Coleoptera that live in microhabitats associated with standing trees (Lindgren 1983). See Webster et al. (in press) for details of the methods used to deploy the Lindgren 12-funnel traps and of sample collection. A description of the habitat was recorded for all specimens collected during this survey. Locality and habitat data are presented exactly as on labels for each record. This information, as well as additional collecting notes, is summarized and discussed in the collection and habitat data section for each species.

### Specimen preparation and determination

Keys in Downie and Arnett (1996) and Majka (2010) were used to determine specimens of Eucinetidae. Klausnitzer (1976) and Epler (2010) were consulted for determining Scirtidae specimens. Specimens were compared with material in the Canadian National Collection of Insects for confirmation.

### Distribution

Distribution maps, created using ArcMap and ArcGIS, are presented for each species in New Brunswick. Every species is cited with current distribution in Canada and Alaska, using abbreviations for the state, provinces, and territories. New records for New Brunswick are indicated in bold under Distribution in Canada and Alaska. The following abbreviations are used in the text:

**Table:** 

**AK**	Alaska	**MB**	Manitoba
**YT**	Yukon Territory	**ON**	Ontario
**NT**	Northwest Territories	**QC**	Quebec
**NU**	Nunavut	**NB**	New Brunswick
**BC**	British Columbia	**PE**	Prince Edward Island
**AB**	Alberta	**NS**	Nova Scotia
**SK**	Saskatchewan	**NF & LB**	Newfoundland and Labrador*

*Newfoundland and Labrador are each treated separately under the current Distribution in Canada and Alaska.

Acronyms of collections examined or where specimens referred to in this study reside are as follows:

**AFC** Atlantic Forestry Centre, Natural Resources Canada, Canadian Forest Service, Fredericton, New Brunswick, Canada

**CNC** Canadian National Collection of Insects, Arachnids and Nematodes, Agriculture and Agri-Food Canada, Ottawa, Ontario, Canada

**NBM** New Brunswick Museum, Saint John, New Brunswick, Canada

**RWC** Reginald P. Webster Collection, Charters Settlement, New Brunswick, Canada

## Results

### Species accounts

All records below are species newly recorded for New Brunswick, Canada unless noted otherwise (additional records). Species followed by ** are newly recorded from the Maritime provinces of Canada; species followed by *** are newly recorded from Canada.

The classification of the Eucinetidae follows Young (2002a). The classification of the Scirtidae follows Young (2002b) and Bouchard et al. (2011).

### Family Eucinetidae Lacordaire, 1857

The Eucinetidae (the plate-thigh beetles) have greatly expanded metathoracic coxal plates that conceal much of the first abdominal segment and the metathoracic legs. Adults live in various kinds of litter or under fungus-covered bark (Young 2002a). Larvae are mycophagous and feed on a variety of fungi (Weiss and West 1921; Wheeler and Hoebeke 1984). Campbell (1991a) reported *Eucinetus haemorrhoidalis* (Germar) and *Nycteus testaceus* (LeConte) from New Brunswick. Majka (2010) reviewed the Eucinetidae of the Maritime provinces and reported *Eucinetus morio* LeConte as new but questioned the validity of the *Nycteus testaceus* record from New Brunswick due to lack of a supporting voucher and other published records. Here, we report two additional species, *Nycteus oviformis* (LeConte) and *Nycteus punctulatus* (LeConte) for the province and confirm the presence of *Nycteus testaceus*. *Nycteus oviformis* (LeConte) is newly recorded from the Maritime provinces.

#### Eucinetus haemorrhoidalis

(Germar, 1818)

http://species-id.net/wiki/Eucinetus_haemorrhoidalis

Map 1

##### Material examined.

**Additional New Brunswick records. Madawaska Co.**, Loon Lake, 236 m elev., 47.7839°N, 68.3943°W, 21.VII.2010, R. P. Webster, boreal forest, small lake surrounded by sedges, treading sedges and grasses near *Myrica gale* bushes into water (1, NBM). **Queens Co.**, Cranberry Lake P.N.A. (Protected Natural Area), 46.1125°N, 65.6075°W, 12–21.V.2009, 21–27.V.2009, 5–11.VI.2009, R. Webster & M.-A. Giguère, old red oak forest, Lindgren funnel trap (1,RWC). **Sunbury Co.**, Acadia Research Forest, 45.9816°N, 66.3374°W, 12.V.2007, R. P. Webster, 8.5 year-old regenerating mixed forest, sifting moss and litter (2, RWC); same locality but 45.9866°N, 66.3841°W, 19–25.V.2009, R. Webster & M.-A. Giguère, red spruce forest with red maple and balsam fir, Lindgren funnel traps (2, RWC). **York Co.**, 15 km W of Tracy off Rt. 645, 45.6848°N, 66.8821°W, 19–25.V.2009, 1–8.VI.2009, R. Webster & M.-A. Giguère, old red pine forest, Lindgren funnel traps (2, AFC, RWC); Charters Settlement, 45.8267°N, 66.7343°W, 30.IV.2005, R. P. Webster, *Carex* marsh, in sphagnum in *Carex* hummock (2, RWC); same locality and collector but 45.8331°N, 66.7410°W, 14.IV.2006, mixed forest, in litter and sphagnum (1, RWC).

**Map 1.**
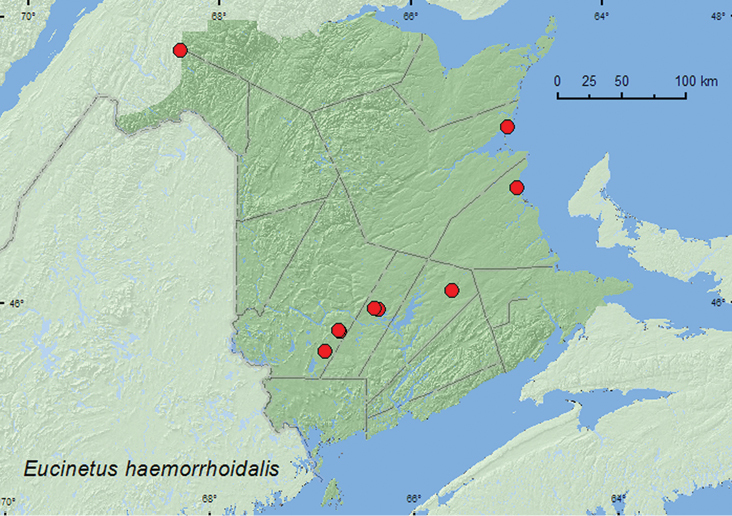
Collection localities in New Brunswick, Canada of *Eucinetus haemorrhoidalis*.

##### Collection and habitat data.

This species was collected along a lake margin, and in a *Carex* marsh, an old red oak (*Quercus rubra* L.) forest, a mature red spruce (*Picea rubens* Sarg.) forest, an old red pine (*Pinus resinosa* Ait.) forest, a mature mixed forest, and an 8.5-year-old regenerating mixed forest. Adults were collected by treading sedges (*Carex* sp.) and grasses along a lake margin, sifting moss and leaf litter, and sifting sphagnum from a *Carex* hummock in a *Carex* marsh. This species was capable of jumping out of a 15 cm high sifting box. This species was also captured in Lindgren funnel traps. Adults were collected during April, May, June, and July.

##### Distribution in Canada and Alaska.

NT, BC, AB, SK, MB, ON, QC, NB, NS, PE (Campbell 1991a; Majka 2010). This species was recorded from New Brunswick by Campbell (1991a) based on specimens collected in Kouchibouguac National Park (Kent Co.) and Tabusintac (Northumberland Co.).

#### Eucinetus morio

LeConte, 1853

http://species-id.net/wiki/Eucinetus_morio

Map 2

##### Material examined.

**Additional New Brunswick records. Carleton Co.**, Two Mile Brook Fen, 46.3619°N, 67.6733°W, 6.V.2005, R. Webster & M.-A. Giguère, cedar forest/swamp, in moist sphagnum (1, RWC); Jackson Falls, Bell Forest, 46.2200°N, 67.7231°W, 8.VIII.2006, R. P. Webster, mature hardwood forest, on polypore fungi on dead standing beech (1, NBM); same locality and forest type but 4–12.VI.2008, 27.VI–5.VII.2008, 12–19.VII.2008, R. P. Webster, mature hardwood forest, Lindgren funnel traps (5, AFC, RWC) ); same locality and habitat data but 28.IV-9.V.2009, 9–14.V.2009, 14–20.V.2009, 20–26.V.2009, 31.VII–7.VIII.2009, R. Webster & M.-A. Giguère, Lindgren funnel traps (7, AFC, RWC). **Charlotte Co.**, 10 km NW of New River Beach, 45.2110°N, 66.6170°W, 31.V–15.VI.2010, 16–26.VII.2010, R. Webster & C. MacKay, old growth eastern white cedar forest, Lindgren funnel traps (2, AFC). **Queens Co.**, Cranberry Lake P.N.A., 46.1125°N, 65.6075°W, 12–21.V.2009, 21–27.V.2009, 5–11.VI.2009, 28.VII-6.VIII.2009, R. Webster & M.-A. Giguère, old red oak forest, Lindgren funnel traps (4, AFC, RWC). **Restigouche Co.**, Dionne Brook P.N.A., 47.9064°N, 68.3441°W, 31.V-15.VI.2011, M. Roy & V. Webster, old-growth white spruce and balsam fir forest, Lindgren funnel trap (1, NBM). **Sunbury Co.**, Acadia Research Forest, 45.9866°N, 66.3841°W, 19–25.V.2009, 2–9.VI.2009, 16–24.VI.2009, 24–30.VI.2009, 29.VII-4.VIII.2009, R. Webster & M.-A. Giguère, red spruce forest with red maple and balsam fir, Lindgren funnel traps (8, AFC). **York Co.**, Charters Settlement, 45.8286°N, 66.7365°W, 10.VII.2005, R. P. Webster, mature red spruce and cedar forest, in powdery slime mould (1, NBM); same locality but 45.8331°N, 66.7410°W, 17.VIII.2008, R. P. Webster, mature red spruce forest, in polypore fungi on dead standing *Populus* sp. (1, RWC); 15 km W of Tracy off Rt. 645, 45.6848°N, 66.8821°W, 1–8.VI.2009, 28.VI-7.VII.2009, 29.VII-4.VIII.2009, R. Webster & M.-A. Giguère, old red pine forest, Lindgren funnel traps (6, AFC); 14 km WSW of Tracy, S of Rt. 645, 45.6741°N, 66.8661°W, 10–26.V.2010, 16–30.VI.2010, R. Webster & C. MacKay, old mixed forest with red and white spruce, red and white pine, balsam fir, eastern white cedar, red maple, and *Populus* sp., Lindgren funnel traps (2, AFC).

**Map 2.**
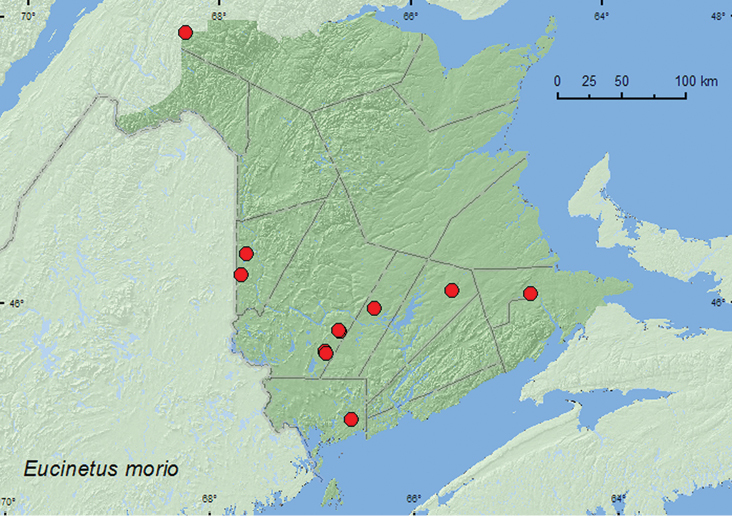
Collection localities in New Brunswick, Canada of *Eucinetus morio*.

##### Collection and habitat data.

*Eucinetus morio* was found in a variety of forest types in New Brunswick, including mature hardwood forests, an old red oak forest, old and mature mixed forests, an old-growth white spruce (*Picea glauca* (Moench) Voss) and balsam fir (*Abies balsamea* (L.) Mill.) forest, eastern white cedar (*Thuja occidentalis* L.) forests, a mature (110-year-old) red spruce forest, and an old red pine forest. Most specimens were captured in Lindgren funnel traps deployed in the above forest types. Specimens with specific habitat data were collected from moist sphagnum (in eastern white cedar swamp), on polypore fungi on dead standing American beech (*Fagus grandifolia* Ehrh.) and a dead standing *Populus* sp., and in powdery slime mold at the base of a tree. Lawrence and Newton (1980) reported the slime mold, *Stemonitis axifera* (Bull.) as a host for this species, and Weiss and West (1921) reported it from a *Trichia* sp. (Trichiaceae). This species has an amazing jumping ability, and adults often jumped out of a 15 cm high sifting box. Adults were collected during May, June, July, and August.

##### Distribution in Canada and Alaska.

ON, QC, NB, NS (Campbell 1991a, Majka 2010). Majka (2010) newly reported *Eucinetus morio* from New Brunswick based on one specimen collected by P. Maltais in Moncton (Westmorland Co.). This is the most common species of Eucinetidae in New Brunswick.

#### Nycteus oviformis

(LeConte, 1866)**

http://species-id.net/wiki/Nycteus_oviformis

Map 3

##### Material examined.

**New Brunswick, Queens Co.**, Cranberry Lake P.N.A, 46.1125°N, 65.6075°W, 18–31.VIII.2011, M. Roy & V. Webster, old red oak forest, Lindgren funnel trap (1, RWC). **Restigouche, Co.**, Dionne Brook P.N.A., 47.9064°N, 68.3441°W, 27.VI-14.VII.2011, 14–28.VII.2011, M. Roy & V. Webster, old-growth white spruce and balsam fir forest, Lindgren funnel traps (2, RWC). **York Co.**, Charters Settlement, 45.8430°N, 66.7275°W, 30.VI.2008, R. P. Webster, regenerating mixed forest, brushy opening, sweeping foliage (1, RWC).

**Map 3.**
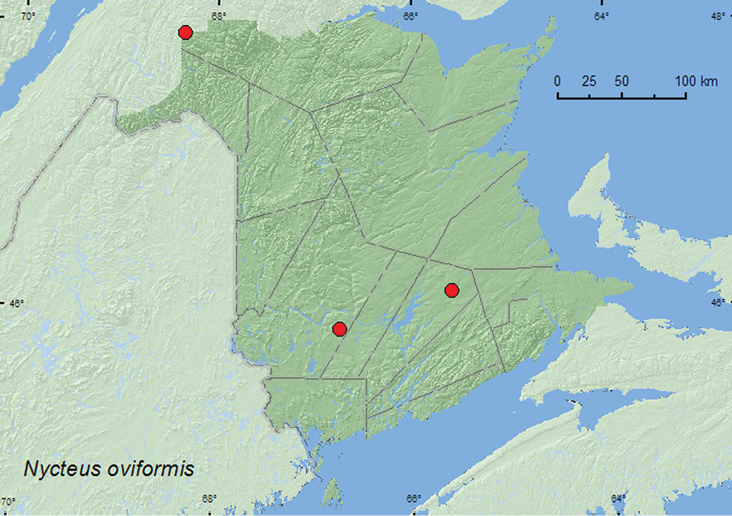
Collection localities in New Brunswick, Canada of *Nycteus oviformis***.**

##### Collection and habitat data.

Adults were captured in Lindgren funnel traps deployed in an old red oak forest and an old-growth white spruce and balsam fir forest. One individual was swept from foliage in a brushy opening of a regenerating (15-year-old) mixed forest. Adults were captured during June, July, and August.

##### Distribution in Canada and Alaska.

MB, **NB** (Campbell 1991a).

#### Nycteus punctulatus

(LeConte, 1875)

http://species-id.net/wiki/Nycteus_punctulatus

Map 4

##### Material examined.

**New Brunswick, Queens Co.**, Cranberry Lake P.N.A, 46.1125°N, 65.6075°W, 18–25.VI.2009, 25.VI-1.VII.2009, 1–10.VII.2009, 28.VII–6.VIII.2009, R. Webster & M.-A. Giguère, old red oak forest, Lindgren funnel traps (5,RWC); same locality data and forest type, 7–13.VII.2011, 18–31.VIII.2011, M. Roy & V. Webster, Lindgren funnel traps (2, NBM, RWC). **Restigouche Co.**, Dionne Brook P.N.A., 47.9030°N, 68.3503°W, 14–28.VII.2011, 28.VII-9.VIII.2011, 9–23.VIII.2011, M. Roy & V. Webster, old-growth northern hardwood forest, Lindgren funnel traps (7, AFC, NBM, RWC); same locality and collectors but 47.9064°N, 68.3441°W, 27.VI-14.VII.2011, 9–23.VIII.2011, old-growth white spruce and balsam fir forest, Lindgren funnel traps (7, AFC, NBM, RWC).

**Map 4.**
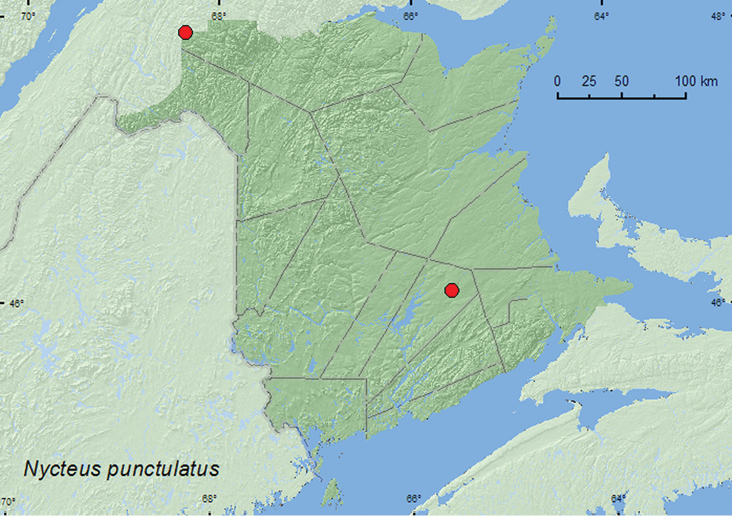
Collection localities in New Brunswick, Canada of *Nycteus punctulatus*.

##### Collection and habitat data.

The New Brunswick adults were captured in Lindgren funnel traps deployed in an old red oak stand, an old-growth northern hardwood forest with sugar maple (*Acer saccharum* Marsh.) and yellow birch (*Betula alleghaniensis* Britt.), and in an old-growth white spruce and balsam fir forest. Adults were collected during June, July, and August.

##### Distribution in Canada and Alaska.

YK, BC, AB, SK, MB, ON, QC, **NB**, NS (Campbell 1991a; Majka 2010).

#### Nycteus testaceus

(LeConte, 1866)

http://species-id.net/wiki/Nycteus_testaceus

Map 5

##### Material examined.

**Additional New Brunswick records, Carleton Co.**, Jackson Falls, Bell Forest, 46.2199°N, 67.7232°W**,** 13.VIII.2007, R. P. Webster, hardwood forest, on gilled mushrooms (4, RWC); same locality but 46.2210°N, 67.7210°W, 25.VII.2007, R. P. Webster, hardwood forest, u.v. light (1, RWC). **Queens Co.**, Cranberry Lake P.N.A, 46.1125°N, 65.6075°W, 25.VI–1.VII.2009, R. Webster & M.-A. Giguère, old red oak forest, Lindgren funnel trap (1,RWC). **York Co.**, Charters Settlement, 45.8395°N, 66.7391°W, 23.VII.2007, 7.IX.2007, R. P. Webster, mixed forest, u.v. light (2, RWC).

**Map 5.**
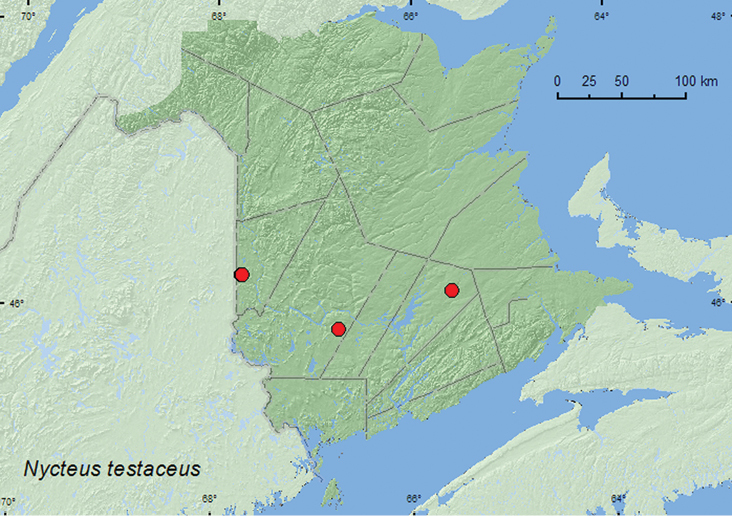
Collection localities in New Brunswick, Canada of *Nycteus testaceus*.

##### Collection and habitat data.

*Nycteus testaceus* was collected from gilled mushrooms on the forest floor of a hardwood forest, and at an ultraviolet light in a mixed forest and a hardwood forest. One individual was captured in a Lindgren funnel trap deployed in an old red oak forest. This species was capable of jumping out of a 15 cm high sifting box, resulting in the loss of a number of specimens collected from gilled mushrooms. Adults were captured during July, August, and September.

##### Distribution in Canada and Alaska.

NT, BC, AB, SK, MB, ON, QC, NB (Campbell 1991a). Campbell (1991a) reported this species from New Brunswick. However, Majka (2010) could not find any specimens or published source to support the record and, therefore, considered the status of this species in the province as hypothetical. The above records confirm the presence of this species for New Brunswick.

### Family Scirtidae Fleming, 1821

The Scirtidae (the marsh beetles), as their common name implies, are associated with marshes and other kinds of wetlands (Young 2002b). Larvae are generally aquatic and frequent stagnant and flowing waters such as forest pools, streams, rivers, various marsh types, and sphagnum bogs (Young 2002b). The North American species are badly in need of revision, especially the Genus *Cyphon*. Tetrault (1967), in an unpublisded Ph.D. dissertation revised the North American species of the family and described several new *Cyphon* species. However, since the dissertation was never published these names are not available. Later, Klausnitzer (1976) and Young and Stribling (1990) described some other North American species of *Cyphon*. Campbell (1991b) reported seven species of Scirtidae from New Brunswick. However, there have been no recent treatments of this family for New Brunswick or the Maritime provinces. Here, we report five species new to the province (Table 1). *Sarabandus robustus* (LeConte) is newly recorded from Canada; *Cyphon ruficollis* (Say), *Prionocyphon discoideus* (Say), and *Sacodes pulchella* (Guérin-Méneville) are added to the faunal list of the Maritime provinces.

**Table 1.** Species of Eucinetidae and Scirtidae recorded from New Brunswick, Canada.

**Family Eucinetidae Lacordaire**
*Eucinetus haemorrhoidalis* (Germar)
*Eucinetus morio* LeConte
*Nycteus oviformis* (LeConte)**
*Nycteus punctulatus* (LeConte)*
*Nycteus testaceus* (LeConte)
**Family Scirtidae Fleming**
**Subfamily Scirtinae Fleming**
*Cyphon collaris* (Guérin-Méneville)
*Cyphon neovariabilis* Klausnitzer
*Cyphon obscurus* (Guérin-Méneville)
*Cyphon ruficollis* (Say)**
*Cyphon variabilis* (Thunberg)
*Elodes maculicollis* Horn*
*Microcara explanata* LeConte
*Prionocyphon discoideus* (Say)**
*Prionocyphon limbatus* LeConte
*Sacodes pulchella* (Guérin-Méneville)**
*Sarabandus robustus* (LeConte)***
*Scirtes tibialis* Guérin-Méneville

**Notes:** *New to province, **New to Maritime provinces, ***New to Canada

### Subfamily Scirtinae Fleming, 1821

#### Cyphon ruficollis

(Say, 1825)**

http://species-id.net/wiki/Cyphon_ruficollis

Map 6

##### Material examined. 

**New Brunswick, York Co.**, Fredericton, Odell Park, 45.9570°N, 66.6695°W, 19.VI.2005, R. P. Webster, mixed forest margin, beating foliage (1, RWC).

**Map 6.**
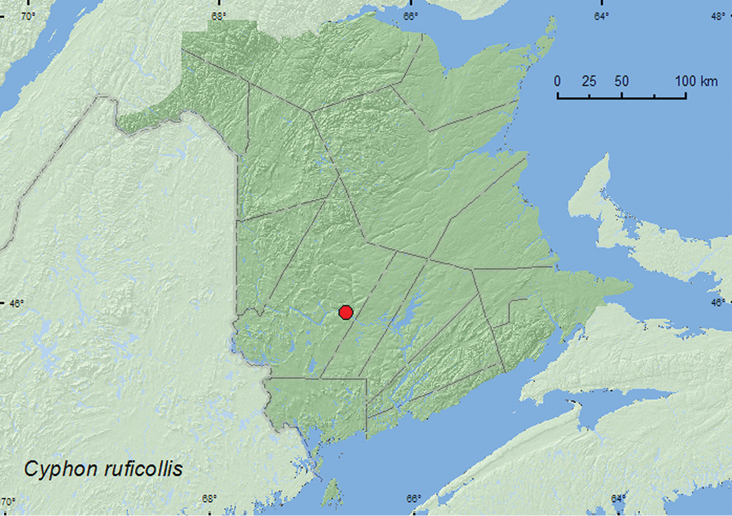
Collection localities in New Brunswick, Canada of *Cyphon ruficollis*.

##### Bionomic notes.

One individual was collected from foliage along a mixed forest margin during June.

##### Distribution in Canada and Alaska.

ON, QC, **NB** (Campbell 1991b).

#### Elodes maculicollis

Horn, 1880

http://species-id.net/wiki/Elodes_maculicollis

Map 7

##### Material examined.

**New Brunswick, York Co.**, Charters Settlement, 45.8456°N, 66.7267°W, 16.V.2011, R. P. Webster, beaver dam among sticks and grass litter near overflow area of dam (near flowing water) (4, RWC).

**Map 7.**
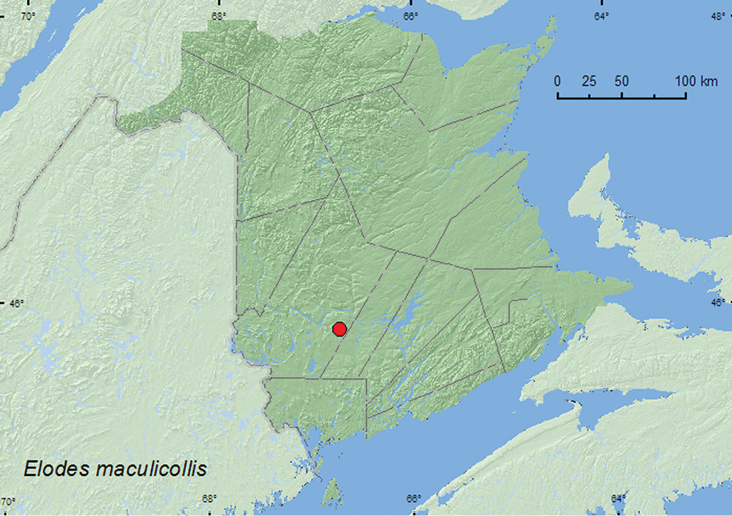
Collection localities in New Brunswick, Canada of *Elodes maculicollis*.

##### Collection and habitat data.

Adults were collected during May inside a beaver (*Castor canadensis* Kuhl) dam near an overflow area in the dam.

##### Distribution in Canada and Alaska.

QC, **NB**, NS, NF (Campbell 1991b).

#### Prionocyphon discoideus

(Say)**

http://species-id.net/wiki/Prionocyphon_discoideus

Map 8

##### Material examined.

**New Brunswick, Carleton Co.**, Jackson Falls, Bell Forest, 46.2200°N, 67.7231°W, 8–16.VI.2009, R. Webster & M.-A. Giguère, mature hardwood forest, Lindgren funnel trap (1, AFC, RWC). **Restigouche, Co.**, Dionne Brook P.N.A., 47.9064°N, 68.3441°W, 14–28.VIII.2011, M. Roy & V. Webster, old-growth white spruce and balsam fir forest, Lindgren funnel trap (1, NBM). **Queens Co.**, Cranberry Lake P.N.A, 46.1125°N, 65.6075°W, 11–18.VI.2009, 18–25.VI.2009, 25.VI-1.VII.2009, R. Webster & M.-A. Giguère, old red oak forest, Lindgren funnel traps (5, AFC, RWC); Grand Lake Meadows P.N.A., 45.8227°N, 66.1209°W, 19–31.V.2010, 15–29.VI.2010, R. Webster & C. MacKay, old silver maple forest with green ash and seasonally flooded marsh, Lindgren funnel traps (2, AFC, RWC). **Sunbury Co.**, Acadia Research Forest, 45.9866°N, 66.3841°W, 24–30.VI.2009, R. Webster & M.-A. Giguère, red spruce forest with red maple and balsam fir, Lindgren funnel trap (1, AFC). **York Co.**, Charters Settlement, 45.8395°N, 66.7391°W, 28.VI.2005, R. P. Webster, mixed forest, u.v. light (1, RWC).

**Map 8.**
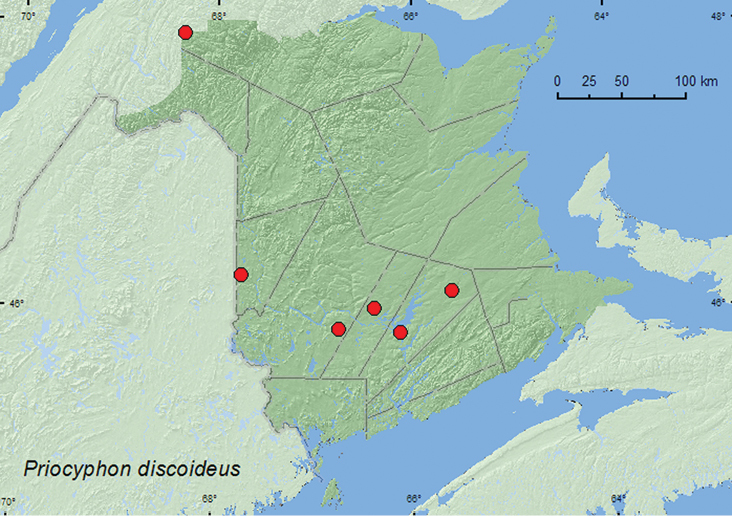
Collection localities in New Brunswick, Canada of *Prionocyphon discoideus*.

##### Collection and habitat notes.

*Prionocyphon discoideus* was captured in Lindgren funnel traps deployed in a variety of deciduous and coniferous forest types in New Brunswick. These included mature mixed forests, an old red oak forest, an old silver maple forest near a seasonally flooded marsh, a red spruce forest and an old-growth white spruce and balsam fir forest. One individual was collected at an ultraviolet light. Adults were captured during May, June, July, and August.

##### Distribution in Canada and Alaska.

ON, QC, **NB** (Campbell 1991b).

#### Sacodes pulchella

(Guérin-Méneville, 1843)**

http://species-id.net/wiki/Sacodes_pulchella

Map 9

##### Material examined.

**New Brunswick, Carleton Co.**, Bell Forest, 46.2200°N, 67.7231°W, 21–28.VI.2009, 19–31.VII.2009, R. Webster & M.-A. Giguère, mature hardwood forest, Lindgren funnel traps (2, AFC, RWC). **Queens Co.**, Cranberry Lake P.N.A, 46.1125°N, 65.6075°W, 25.VI–1.VII.2009, 1–10.VII.2009, 15–21.VII.2009, 21–28.VII.2009, R. Webster & M.-A. Giguère, old red oak forest, Lindgren funnel traps (6, AFC, RWC); same locality data and forest type, 13–20.VII.2011, M. Roy & V. Webster, Lindgren funnel trap (1, NBM). **Restigouche Co.**, Dionne Brook P.N.A., 47.9030°N, 68.3503°W, 27.VI-14.VII.2011, M. Roy & V. Webster, old-growth northern hardwood forest, Lindgren funnel trap (2, RWC). **York Co.**, 15 km W of Tracy off Rt. 645, 45.6848°N, 66.8821°W, 16–30.VI.2010, R. Webster & C. MacKay, old red pine forest, Lindgren funnel trap (1, RWC).

**Map 9.**
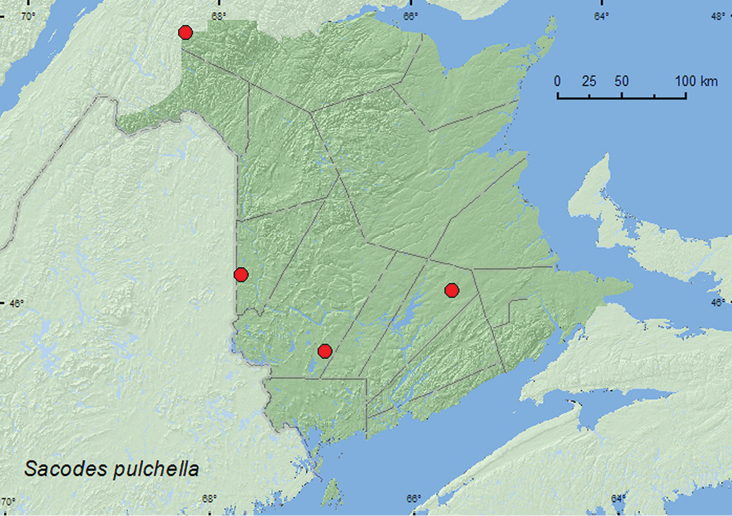
Collection localities in New Brunswick, Canada of *Sacodes pulchella*.

##### Collection and habitat data.

This species was captured in Lindgren funnel traps deployed in a mature hardwood forest with sugar maple, American beech, and white ash (*Fraxinus americana* L.), an old red oak forest, an old-growth northern hardwood forest with sugar maple and yellow birch, and an old red pine forest. Adults were captured during June and July.

##### Distribution in Canada and Alaska.

ON, **NB** (Campbell 1991b).

#### Sarabandus robustus

(LeConte, 1875)***

http://species-id.net/wiki/Sarabandus_robustus

Map 10

##### Material examined.

**Canada, New Brunswick, Charlotte Co.**, near New River, 45.2143°N, 66.6001°W, 2,VI.2006, R. P. Webster, eastern white cedar swamp, in moss and leaf litter (1, RWC).

**Map 10.**
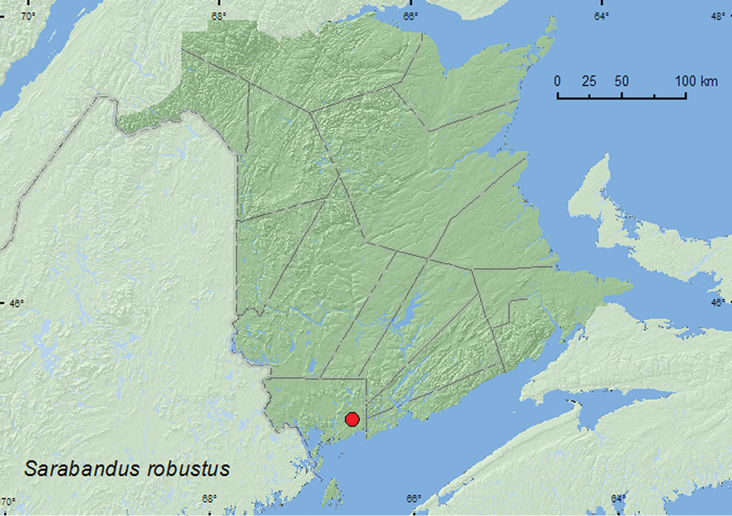
Collection localities in New Brunswick, Canada of *Sarabandus robustus*.

##### Collection and habitat data.

The sole New Brunswick specimen was sifted from moss and leaf litter in an eastern white cedar swamp during early June.

##### Distribution in Canada and Alaska.

(new Canadian record). This species is known from Massachusetts south to Florida (Young 2002a; Epler 2010).

## Supplementary Material

XML Treatment for Eucinetus haemorrhoidalis

XML Treatment for Eucinetus morio

XML Treatment for Nycteus oviformis

XML Treatment for Nycteus punctulatus

XML Treatment for Nycteus testaceus

XML Treatment for Cyphon ruficollis

XML Treatment for Elodes maculicollis

XML Treatment for Prionocyphon discoideus

XML Treatment for Sacodes pulchella

XML Treatment for Sarabandus robustus
